# Decellularised Human Umbilical Artery as a Vascular Graft Elicits Minimal Pro-Inflammatory Host Response Ex Vivo and In Vivo

**DOI:** 10.3390/ijms22157981

**Published:** 2021-07-26

**Authors:** Alexander Høgsted Ahlmann, Shu Fang, Sussi Bagge Mortensen, Line Weis Andersen, Pernille Gejl Pedersen, Johanne Juel Callesen, Sara Thornby Bak, Kate Lykke Lambertsen, Ditte Caroline Andersen

**Affiliations:** 1 DCA-Lab, Department of Clinical Biochemistry and Pharmacology, Odense University Hospital, J.B. Winsløwsvej 25, 5000 Odense C, Denmark; aahlmann@health.sdu.dk (A.H.A.); sfang@health.sdu.dk (S.F.); pgpedersen@health.sdu.dk (P.G.P.); jojujensen@health.sdu.dk (J.J.C.); sabak@health.sdu.dk (S.T.B.); 2 Institute of Clinical Research, University of Southern Denmark, J.B. Winsløwsvej 19, 5000 Odense C, Denmark; Sussi.Bagge.Mortensen@rsyd.dk (S.B.M.); Line.Weis.Andersen@rsyd.dk (L.W.A.); 3 Department of Clinical Immunology, Odense University Hospital, J. B. Winsløwsvej 4, 5000 Odense C, Denmark; 4 Department of Neurobiology, Institute of Molecular Medicine, University of Southern Denmark, J.B. Winsløwsvej 21, 5000 Odense C, Denmark; klambertsen@health.sdu.dk; 5 Department of Neurology, Odense University Hospital, J.B. Winsløwsvej 4, 5000 Odense C, Denmark; 6 BRIDGE—Brain Research—Inter-Disciplinary Guided Excellence, Department of Clinical Research, University of Southern Denmark, J.B. Winsløwsvej 4, 5000 Odense C, Denmark

**Keywords:** tissue engineered vascular grafting, decellularisation, human umbilical artery, innate immunity, macrophage M1 and M2 responses

## Abstract

Small diameter (<6 mm) vessel grafts still pose a challenge for scientists worldwide. Decellularised umbilical artery (dUA) remains promising as small diameter tissue engineered vascular graft (TEVG), yet their immunogenicity remains unknown. Herein, we evaluated the host immune responses, with a focus on the innate part, towards human dUA implantation in mice, and confirmed our findings in an ex vivo allogeneic human setup. Overall, we did not observe any differences in the number of circulating white blood cells nor the number of monocytes among three groups of mice (1) dUA patch; (2) Sham; and (3) Mock throughout the study (day −7 to 28). Likewise, we found no difference in systemic inflammatory and anti-inflammatory cytokine levels between groups. However, a massive local remodelling response with M2 macrophages were observed in the dUA at day 28, whereas M1 macrophages were less frequent. Moreover, human monocytes from allogeneic individuals were differentiated into macrophages and exposed to lyophilised dUA to maximize an eventual M1 response. Yet, dUA did not elicit any immediate M1 response as determined by the absence of CCR7 and CXCL10. Together this suggests that human dUA elicits a minimal pro-inflammatory response further supporting its use as a TEVG in an allogeneic setup.

## 1. Introduction

Cardiovascular diseases (CVDs) are one of the heaviest health burdens globally. Approximately 18 million people died from CVDs in 2016, and about half of them died due to ischemic heart disease (IHD), making it the leading cause of death worldwide [1]. For more than 50 years, scientists have worked on developing tissue engineered vascular grafts (TEVGs), and indeed large diameter (>6 mm) TEVGs are nowadays successfully used for vascular bypass surgery. However, small diameter (<6 mm) TEVGs still remain a major challenge in the field [2], where synthetic, natural and hybrid grafts cause unwanted reactions in the blood-material and tissue-material interfaces leading to thrombosis [3]. As an alternative, decellularised vessels are being explored, since they offer the correct 3D structure and natural extracellular matrix (ECM) composition with several decellularization methods already existing [4,5]. In this regard, we recently showed that human umbilical artery (UA) was successfully decellularised, easily handled by surgeons, and withstood the in vivo blood pressure in a sheep model for carotid bypass [5]. The decellularised human UA holds several advantages over synthetic, autologous or cadaveric allogeneic sources of vascular grafts, providing readily available fresh tissue without invasive collection procedures. Human umbilical cords are considered waste material at hospitals worldwide and can be obtained upon informed consent without substantial ethical concerns. With a length of 30–55 cm, a lumen diameter of 2–4.2 mm and a wall thickness of 212–417 µm, the size of the human UAs from the umbilical cord are well suited as vascular grafts for small diameter TEVGs [6,7] providing a native vascular environment as compared to synthetic alternatives. Decellularised vessels are devoid of native cells while the ECM is preserved [5,8]. This latter notion is indeed important since the ECM influences determination, differentiation, proliferation, survival, polarity, and migration of recellularising cells and, thus, proper vessel functionality [9,10]. Accordingly, the ECM may also evoke inflammatory responses [11], a central issue often neglected when evaluating tissue-engineered material. When using an allogeneic setup, where a decellularised vessel from a diseased or living donor is used, it is important to consider whether the ECM elicits a pro-inflammatory response or a constructive remodelling. Known factors affecting this outcome are: age of the source tissue, the use of chemical crosslinking agents, and the efficiency of the decellularisation [11,12]. Specifically, macrophages, depending on their polarization profile, may be involved [13]. The M1 macrophage phenotype is classically activated and has an unwanted pro-inflammatory nature, whereas the M2 phenotype is alternatively activated and exhibits beneficial anti-inflammatory properties [14,15]. M0 macrophages are considered inactive, but may, upon stimulation, be polarised into M1 or M2 [16,17]. Accordingly, interferon gamma (IFNγ), lipopolysaccharide (LPS), and/or tumour necrosis factor-alpha (TNF-α) dictate M1 polarization while the M2 phenotype can be induced by interleukin (IL)-4, IL-13, or IL-10 [18,19]. Macrophage polarization can be determined by cytokine expression profiles [20]. However, knowledge on host macrophage polarization due to decellularised tissues is still poorly described, and to our knowledge absent with respect to human decellularised umbilical artery (dUA).

We therefore set out to determine whether human dUA evokes in vivo and ex vivo pro-inflammatory responses that may harness their future use as TEVGs.

## 2. Results

### 2.1. Decellularised Umbilical Artery Does Not Elicit Any Major Systemic Inflammatory Responses upon Subcutaneous Implantation in Mice

**Figure 1:**
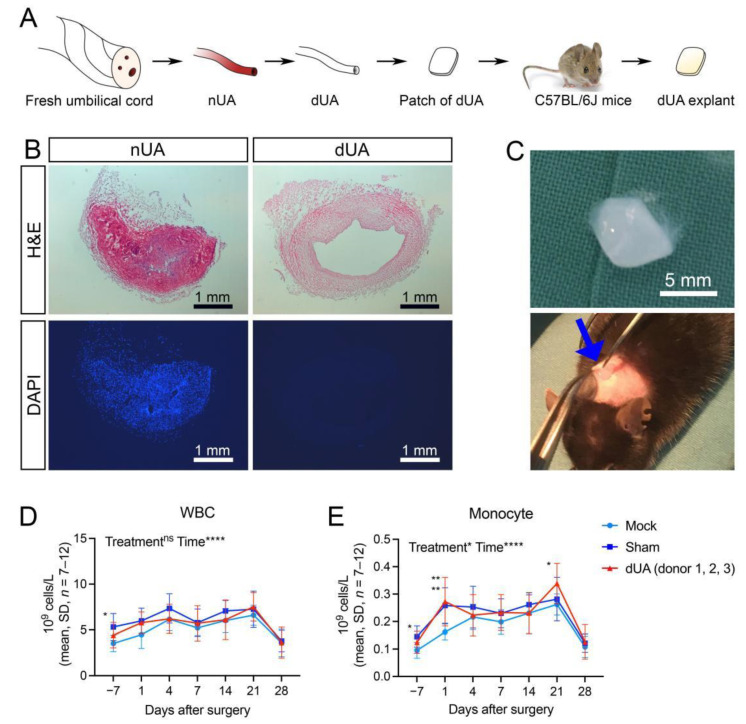
Implantation of decellularised umbilical artery (dUA) in mice. (**A**) Flow chart for the study design for subcutaneous implantation of a human dUA patch into mice. Human native umbilical artery (nUA) was dissected from fresh umbilical cord and decellularised by a 3-day protocol. The dUA was cut into small patches and subcutaneously implanted in mice and explanted after 28 days. (**B**) Haematoxylin and eosin (H&E) and DAPI staining of nUA and dUA, showing efficient decellularisation. (**C**) Representative images of a 5 × 5 mm dUA patch inserted in a subcutaneous dorsal pocket (marked by blue arrow) on a C57BL/6J mouse. (**D**,**E**) White blood cell (WBC) and monocyte count in peripheral blood was monitored 7 days before and at 1, 4, 7, 14, 21, and 28 days after implantation. The effect of treatment (mock, sham and dUA) and time after surgery were analysed by mixed-effects analysis (post-test: Tukey test) for statistics (mock: *n* = 7; sham: *n* = 8; dUA: *n* = 3 (n* = 4)). See also Supplementary Figure S1 for replicate dUA donor measurements. * *p* ≤ 0.05, ** *p* ≤ 0.01, **** *p* ≤ 0.0001, ns *p* > 0.05.

**Figure 2:**
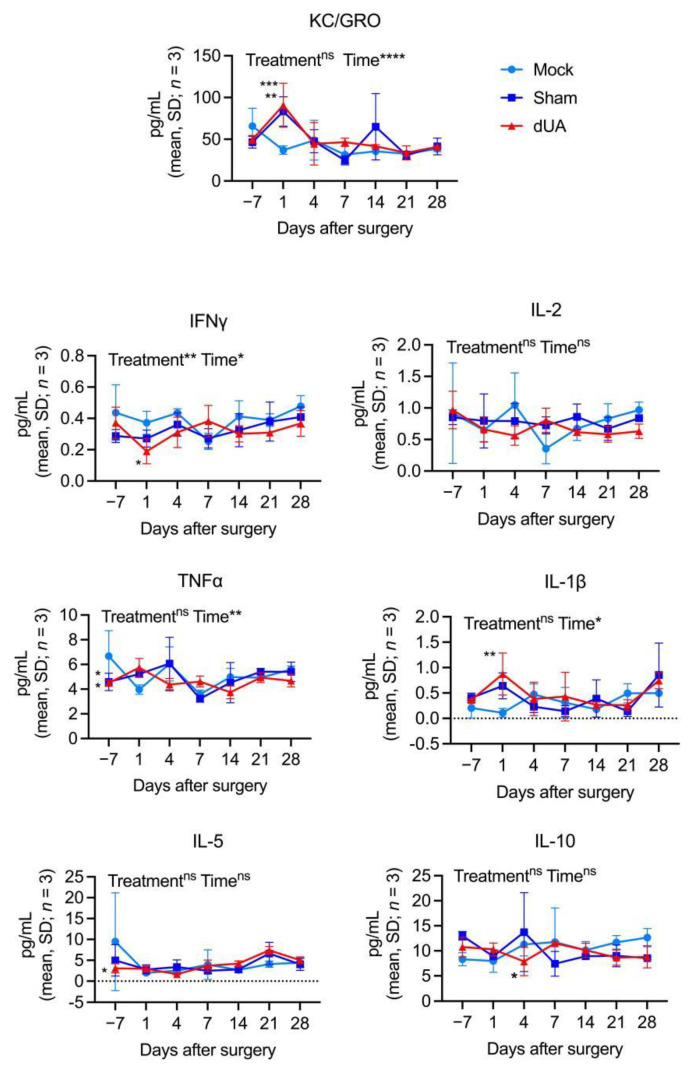
Systemic cytokine levels in mice implanted with human dUA. Cytokine levels were measured 7 days before and at 1, 4, 7, 14, 21, and 28 days after dUA implantation in mice. Each group (mock, sham or dUA) consists of three measurements of randomly pooled plasma from two to four mice from the same group (*n* = 3, n* = 2–4). The effect of treatment (mock, sham and dUA) and time after surgery were analysed by two-way ANOVA (post-test: Tukey test) for statistics. * *p* ≤ 0.05, ** *p* ≤ 0.01, *** *p* ≤ 0.001, **** *p* ≤ 0.0001, ns *p* > 0.05.

Initially, native umbilical arteries (nUA) were dissected from freshly collected umbilical cords (Figure 1A) followed by a 3-day decellularisation procedure [5]. Histological staining validated efficient elimination of cell components and nuclei (Figure 1B), suggesting optimal decellularisation with ECM preservation in agreement with our previous observations [5]. The tubular dUA was then cut longitudinally for preparation of patches (Figure 1A). To mimic the scenario of a graft transplantation, we exploited the mouse subcutaneous implantation model by inserting the dUA patch subcutaneously at the dorsal right side of mice (dUA patch group) (Figure 1C). A total of 12 patches from three different UA donors (four replicates) were implanted into 12 mice. Using this xenogeneic design, we were able to compare both donor and host variability. Moreover, a sham group (*n* = 8) and a mock group (*n* = 7) were included to distinguish surgery specific and dUA non-specific responses. To monitor the general health condition and the occurrence of an inflammatory response systemically, the number of total white blood cells (WBC) and monocytes were counted in peripheral blood during the follow-up for 28 days (Figure 1D,E, Supplementary Figure S1). For both counts, donor replicates (*n* = 4) were similar and suggest that individuals respond equally to dUAs (Supplementary Figure S1). No significant difference was observed in WBC numbers between the three treatment groups (mock, sham and dUA) (Figure 1D). Similarly, monocyte numbers were equal between sham and dUA, although a small increase in monocytes was seen at day 1 in both groups as compared to mock animals (Figure 1E). The latter reflects the general expected response to surgery [21,22]. Moreover, we evaluated the levels of circulating cytokines known to play a role in inflammation. Similar to monocyte numbers, keratinocyte chemoattractant (KC)/human growth-regulated oncogene (GRO) and IL-1β were significantly elevated in both sham and dUA at day 1 (Figure 2), again pointing to a general response to surgery at this early timepoint. Otherwise, cytokine levels (IFNγ, IL-2, TNFα, IL-5, and IL-10) were fairly constant and did not overall differ between treatments (Figure 2). Together these results suggest that efficiently decellularised UA does not evoke major systemic inflammatory and anti-inflammatory host responses upon in vivo implantation.

### 2.2. Human dUA Was Recellularised In Vivo Predominantly by Host Macrophages of the M2 Remodelling Phenotype

**Figure 3:**
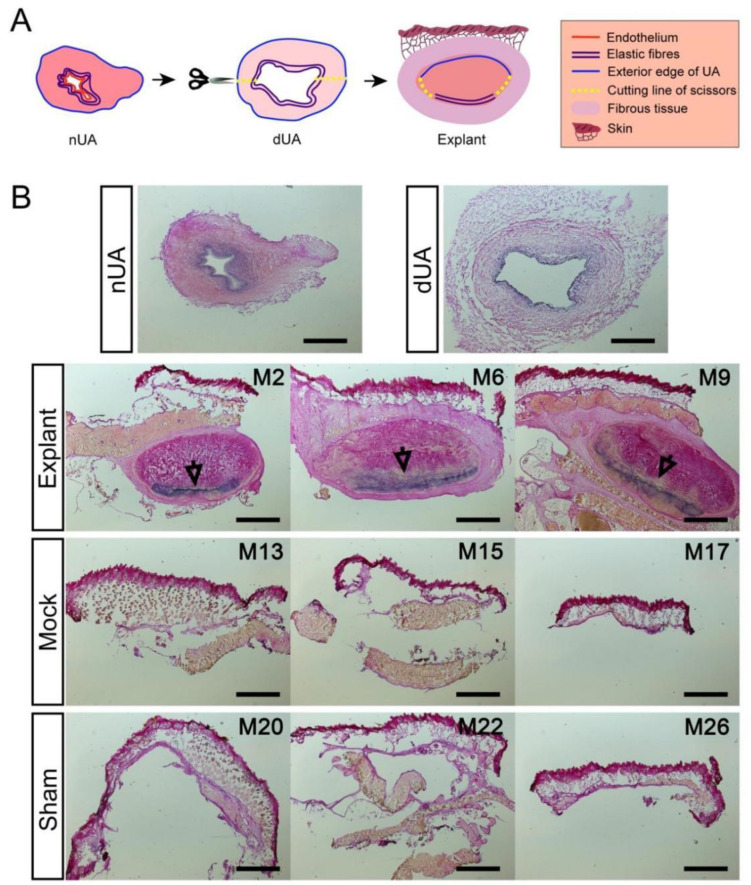
Morphological validation of successful dUA implantation and preservation in explants. (**A**) Schematics of localizing the dUA scaffold in the mouse explant. dUA was cut open into patches and implanted subcutaneously in mice with abluminal side facing the skin of mice. After explantation, the elastic fibres were identified by Miller’s Elastic Van Gieson stain to localise the luminal side of dUA scaffold in explant. (**B**) Miller’s Elastic Van Gieson stain of representative nUA, dUA, and tissue from dUA explant, mock and sham. Dark blue = elastic fibres, pink = mature collagen, yellow = red blood cells, muscle, and other tissues. M2/6/9 and similar present the numbering of mouse individuals. The luminal side of nUA and dUA shows the dark blue stained elastic fibres that were preserved. All dUA scaffolds in the explant displayed a layer of elastic fibres (marked by black arrows) distal to the skin, showing the same direction as they were implanted. No dUA scaffolds are found in tissue from mock and sham groups. Scale bars = 1 mm.

Having evaluated the systemic response in the animals, we next assessed the local response in the graft itself after terminating the experiment at day 28. Before implantation, we observed that elastin fibres at the luminal side of the native UA were preserved in dUA as visualised by Miller’s Elastic Van Gieson staining (Figure 3B). This was used to determine the position of the dUA scaffold in the explanted tissue (Figure 3A,B), which was important for further analysis, but also confirmed the overall preservation of the dUA patch upon in vivo engraftment (Figure 3B).

**Figure 4:**
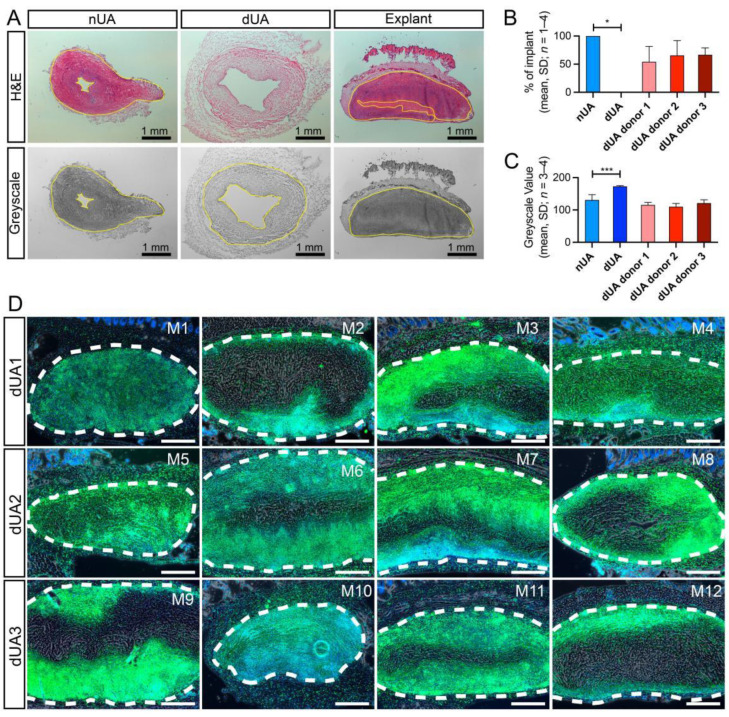
In vivo recellularization of dUA in mice. (**A**) Recellularization of the dUA scaffold in explants was examined by H&E stain. (Upper panel) H&E stain of nUA, dUA, and explant. The area of dUA scaffold showing host cell infiltration (marked with yellow circle) was quantified as area fraction of the total dUA scaffold. (Lower panel) greyscale images converted from H&E images. The mean greyscale value of the dUA scaffold (marked with yellow circle) in explant was quantified by ImageJ (1.52a). The lower mean greyscale value is associated with more recellularization in the area. (**B**) Quantification results of the recellularised area fraction of H&E images of nUA, dUA, and three explant groups according to their dUA donor. (**C**) Quantification results of the mean greyscale value of nUA, dUA, and three explant groups according to their dUA donor. Ordinary one-way ANOVA using Dunnett’s multiple comparisons test was applied for statistics (**B**,**C**). * *p* ≤ 0.05, *** *p* ≤ 0.001. (**D**) Haematopoietic marker CD45 (green) fluorescent stain of all dUA explants from 12 mouse individuals (M1–M12) from three dUA donors. Nuclei are stained by DAPI (blue). Antibody controls did not reveal any non-specific staining (Data not shown). Dotted lines mark the edge of the dUA scaffold. Scale bars = 500 µm.

**Figure 5:**
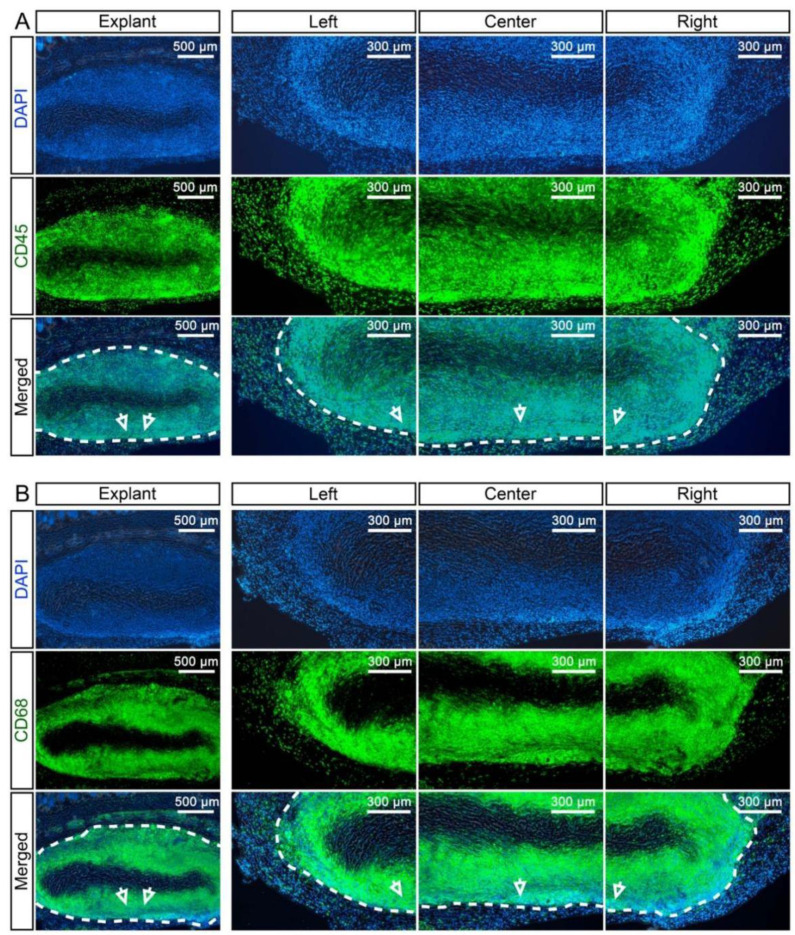
Identification of the cell type invading the dUA upon in vivo implantation. Representative images from fluorescent staining for the (**A**) well known haematopoietic marker (except for erythrocytes) CD45 (green) and (**B**) the macrophage marker CD68 (green) in dUA explants (*n* = 12). Nuclei are stained by DAPI (blue). Antibody controls did not reveal any non-specific staining (data not shown). The arrows mark the same location of the dUA scaffold in different magnifications. Dotted lines mark the edge of the dUA scaffold.

**Figure 6:**
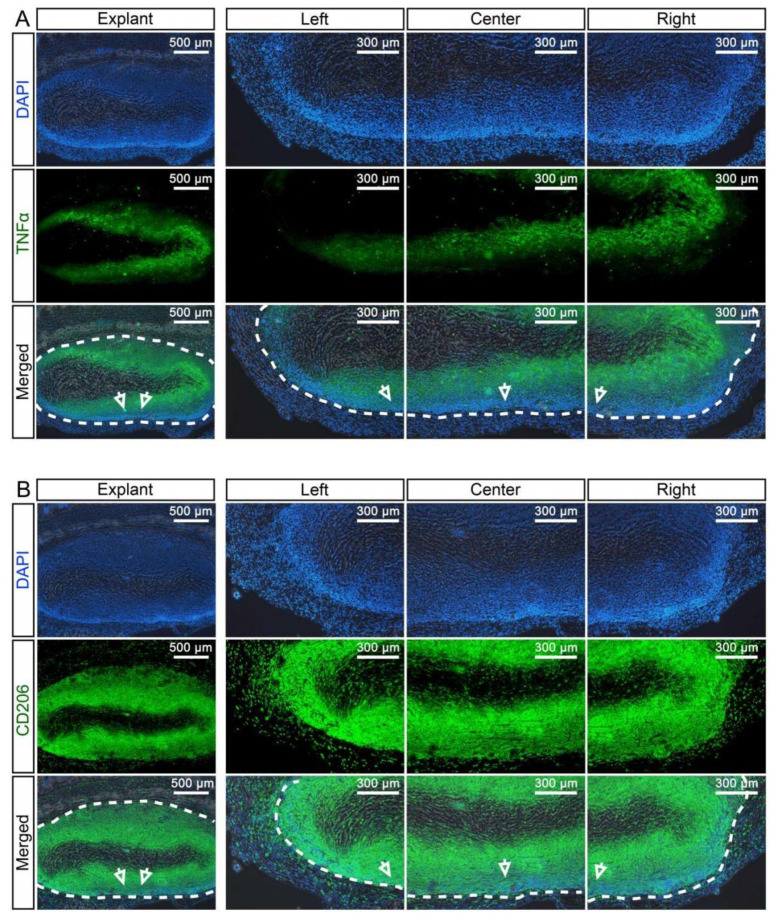
Identification of the macrophage subtype invading the dUA upon in vivo implantation. Representative images from fluorescent staining of the (**A**) M1 macrophage marker TNFα (green) and the (**B**) M2 macrophage marker CD206 (green) in dUA explants (*n* = 12). Nuclei are stained by DAPI (blue). Antibody controls did not reveal any non-specific staining (data not shown). The arrows mark the same location of the dUA scaffold in different magnifications. Dotted lines mark the edge of the dUA scaffold.

Next, we evaluated the degree of host cell recellularization that had occurred in the dUA patches in vivo, using quantifications of cell infiltration area in haematoxylin and eosin (H&E) stained sections of the explants (Figure 4A–C). As such, the explanted dUA displayed recellularization by host cells at 60% of the growth area, when nUA and dUA were defined as 100% and 0%, respectively. Moreover, when considering the replicate (n* = 4) dUAs of the three individual umbilical artery donors, we did not observe any significant difference (Figure 4B). Using a less subjective quantification method where we converted the images measuring mean greyscale values of the degree of recellularization, explants were shown to be almost fully recellularised by host cells (Figure 4C). Using immunofluorescence with detection of CD45, a ubiquitous haematopoietic marker (Figure 4D), we found that the majority of infiltrated cells in the dUA patches were of haematopoietic origin (Figure 4D). In four explants (M2/8/9/12), infiltrating CD45^+^ cells resided only at the margin of the dUA scaffold, whereas the remaining eight explants displayed CD45^+^ cell recellularization throughout the tissue, except for a small non-recellularised area at the centre of the scaffold. As shown by the macrophage marker CD68 stain, the majority of infiltrated immune cells (Figure 5A) were CD68^+^ macrophages (Figure 5B). We next determined whether M1 or M2 macrophages composed the local host macrophage response observed and, therefore, evaluated TNF-α and CD206 expression to further stratify according to M1 and M2 macrophage polarization, respectively (Figure 6). A small area next to the non-recellularised centre of the dUA encompassed some TNF-α positive cells (Figure 6A), whereas the majority of infiltrated cells expressed CD206 (Figure 6B). Moreover, we found substantial numbers of cells throughout most dUA explants expressing the endothelial marker CD31 (Supplementary Figure S2), further reflecting active remodelling [23]. Together, these data indicated that, although an immediate systemic immune response is lacking upon in vivo dUA engraftment, a significant local host macrophage remodelling M2 response occurs throughout the dUA scaffold.

### 2.3. Human Macrophage Polarization Assay Revealed Absence of Pro-Inflammatory Response on Lyophilised and Pulverised dUA during Ex Vivo Scaffold Challenge

**Figure 7:**
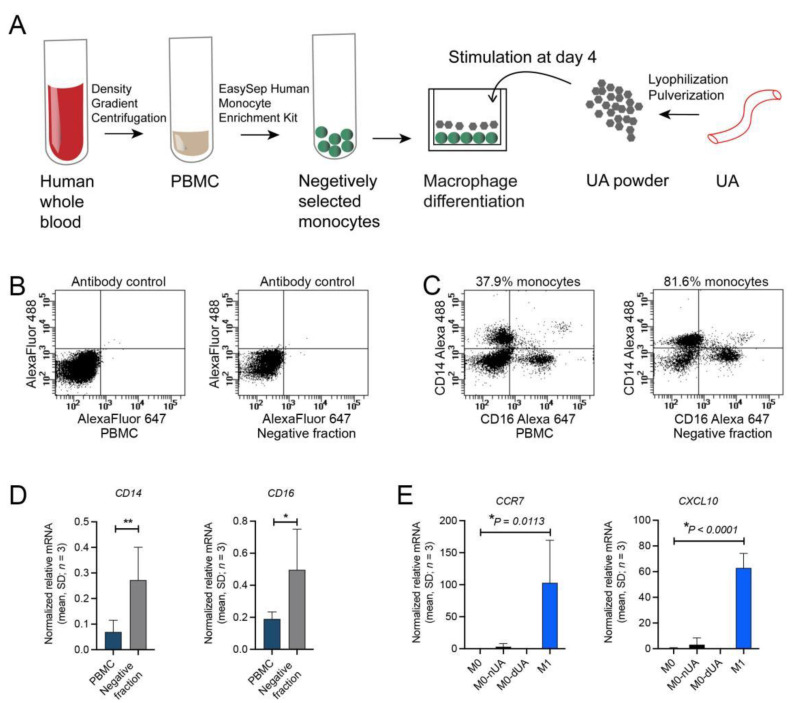
Ex vivo human pro-inflammatory profile of lyophilised human dUA powder. (**A**) Flow chart of human macrophage polarization upon lyophilised and powdered dUA stimulation. Peripheral blood mononuclear cells (PBMCs) were isolated from human whole blood (*n* = 3) after density gradient centrifugation. Human monocytes were isolated in the negative selected fraction and differentiated into M0 macrophages. At day 4, cells were stimulated with either lyophilised/powdered dUA or nUA or LPS/IFNγ for M1 stimulation (*n* = 3). (**B**,**C**) Representative flow cytometry of PBMCs and negative selected monocytes using (**B**) antibody controls or (**C**) CD14 and CD16 antibodies confirming monocyte enrichment. (**D**) Relative quantitative RT-PCR of PBMCs and negative selected monocytes for CD14 and CD16 mRNA expression further supporting monocyte enrichment. Ratio paired t-test (two-tailed) is used for statistical analysis. * *p* ≤ 0.05, ** *p* ≤ 0.01. (**E**) Relative quantitative RT-PCR of the pro-inflammatory M1 markers (CCR7 and CXCL10, see also Supplementary Figure S4) in M0 macrophages, nUA or dUA powder stimulated M0 macrophages (M0-nUA and M0-dUA, respectively), and positive control group (M1) stimulated by LPS and IFNγ. Ordinary one-way ANOVA (post-test: two-stage step-up method of Benjamini, Krieger, and Yekutieli) is used for statistical analysis (*n* = 3). All qRT-PCR data were normalised against multiple stably expressed endogenous controls according to the qbase+ platform (data not shown).

Since our in vivo dUA implantation described above concerned a xenogeneic setup, we next aimed to determine whether an unwanted M1 pro-inflammatory response was present in an allogeneic human setup concerning human dUA. We therefore established a macrophage polarization assay for which human monocytes were isolated from peripheral blood mononuclear cells (PBMCs) of human whole blood, and then differentiated into macrophages before M1 polarization was assessed upon stimulation with dUA, nUA, or a known M1 stimulants (Figure 7A). To maximise the exposure of the possible antigen/immune stimulation to the macrophages, we lyophilised dUA and nUA into powders. Thus, initially we optimised lyophilised dUA pulverization (Supplementary Figure S3) and determined specific M1 markers as compared to M2 markers (Supplementary Figure S4) to discriminate between macrophage subtypes. Flow cytometry of the enriched monocyte PBMC fraction in our approach showed a two-fold enrichment of monocytes expressing CD14, CD16, or both (Figure 7B,C), which was confirmed at the mRNA level (Figure 7D). In a new series of experiments, we then isolated CD14/CD16 positive monocytes from three donors and performed macrophage differentiation. In parallel, we prepared three pairs of allogeneic lyophilised nUAs and dUAs, which were added to the differentiated M0 macrophages together with M1 stimulants as a positive control. Gene expression profiling for the positive M1 control showed a clear and significant increase in the levels of the M1 specific transcripts CCR7 and CXCL10 as compared to unstimulated M0 macrophages (Figure 7E). This confirmed our experimental setup with successful M1 macrophage polarization. In contrast, macrophage exposure to dUA did not evoke any changes in CCR7 and CXCL10 expression (Figure 7E), whereas nUA exposure as expected did have some effect on CCR7 and CXCL10 levels (Figure 7E), although with large variations.

These ex vivo allogeneic human data thus support the in vivo xenogeneic mouse results and indicate that pro-inflammatory responses towards dUA must be limited.

## 3. Discussion

It is well accepted that the success of TEVGs largely depends on the tolerability of the immune system towards the implanted graft [11]. Still, very limited knowledge exists on immune responses directed against materials used for TEVG generation and in particular natural scaffolds, such as decellularised tissues. In the present study, we specifically tested the innate immune response against human dUA and found no major adverse effects. This further supports the future use of dUAs in producing TEVGs for human vascular bypass surgery in patients with limited autologous grafts available.

Specifically, we found that dUA samples implanted in vivo in mice did not cause any major systemic inflammatory responses, since both WBC/monocyte counts and cytokine levels in the blood were fairly constant over the course of the 28-day study. However, whereas our in vitro approach may distinguish macrophage M1 and M2 subtypes, we can only speculate on whether the ratio of M1 to M2 changes within the constant macrophage population observed systemically herein. If the ratio of M1/M2 macrophages increase, then inflammation manifests. Moreover, since we used subcutaneous implantation due to the un-matched sizing of the donor and host, we cannot exclude that insertion directly as a vascular conduit into the blood stream would elicit a systemic response. Previously, we did though test the insertion of 4 cm dUA grafts into sheep carotid artery, with successful blood perfusion [5]. Whereas a lack of sheep antibodies prevented us in that study from assaying the immune response in detail, we did perform blood counts and did not find any major systemic responses towards the inserted dUA, except from day 1 surgery related issues [5] as also noted herein. Recently, others also tested vitrified dUA in a pig model and reported host cell recellularization in dUA. Yet, the immune related process was not assessed [24], which was also the case for previous rodent studies concerning dUA [25]. However, as previously shown by us in a human-sheep setup [5], we also herein observed a local response with CD45^+^ cells invading the dUA. This thus seems independent of the method of insertion [5], and points to a general response. Herein, we found that these CD45^+^ cells mainly comprise macrophages predominantly of the M2 polarization phenotype. However, some evidence of M1 macrophages were observed for dUA in mice and suggest that M1 macrophages are recruited to the inserted dUA although to a much lesser extent than M2 macrophages. This is in agreement with others [15,26,27], where for instance commercially available decellularised mesothelium heart valve products mainly exhibit M2 macrophages recellularisation upon subcutaneous implantation [15]. This stands in contrasts to decellularised porcine small intestinal specimens implanted in a rodent model of body wall repair, where similar numbers of M1 and M2 macrophages were found and which was consistent even after improving the decellularisation method [28]. Indeed, it is likely that the difference in the localization of M1 in the innermost centre of the dUA and the M2 on the dUA outskirts may reflect an initial M1 polarization event when first reaching the dUA, and then a gradual shifting towards the M2 phenotype as exposure continues. Coexistence of M1 and M2 macrophages seems to be a common occurrence, more akin to shifting a balance, than two distinct responses [19]. However, if present in high numbers, M1 macrophages may cause injurious effects such as those observed for the vascular bed in human cardiac allografts where also a prothrombotic environment is mediated [29]. Moreover, M1 macrophages induce intima hyperplasia through the recruitment of smooth muscle cells [30]. In contrast to these M1 effects, M2 polarised macrophages secrete high levels of anti-inflammatory factors and are mainly associated with tissue remodelling [15]. Thus, the presence of high numbers of M2 macrophages in our implanted dUA seem beneficial and supports the idea of using the dUA for TEVG generation. This is further underscored by our results from the human macrophage polarization assay, where human dUA did not evoke M1 polarization of primary derived human macrophages. However, due to the fundamental discrepancy that *in vitro* activated M1 macrophages are not equivalent to classically activated macrophages, and *in vitro* activated M2 macrophages are not equivalent to alternatively activated macrophages, we cannot be certain that the M1 surface marker identified in vitro directly reflects the in vivo situation. Since, these data represent an in vivo human-to-mouse setup, they may possess distinct regenerative characteristics as compared to a human-to-human setup and our experiments using a rodent model may not entirely mimic the conditions of a transplantation in humans. Yet, we did not observe any innate immune response upon ex vivo human-to-human. Thus, our previous and present data suggest that a next step of subcutaneous dUA implantation into humans may be safe at least with respect to the innate immune response. In this regard it is also worth noting that aldehyde fixed human umbilical vein, which include all the cell constituents in addition to the ECM scaffold, already has been tested rigorously in large diameter constructions in humans [31,32,33]. Previous [5] and present data thus suggest that our dUA are efficiently decellularised with preservation of ECM, but without M1 inducing determinants that may otherwise represent ineffective decellularisation [28].

Although our results do not reflect major inflammatory processes related to macrophages, we cannot exclude M1 macrophage mediated acute inflammation at an early phase after implantation. Stimulators that are related to the decellularisation protocol, such as residual cellular components, as we observed in dUA in our previous study [5], damaged nucleic acid fragments, and the ECM with damaged ultrastructure and modifications of secondary conformations could lead to immune response after implantation. Although the decellularisation method we used seems to be optimal as evaluated by a range of ex vivo tests [5], we still need to test and evaluate the immunogenic response caused by all these potential decellularisation artefacts. Yet, considering our perspective, implants are intended to persist in vivo for a very long period, we are currently mainly concerned about late (>28 days) immune responses. It is also important to consider that early M1 responses often precede M2 regenerative remodelling and, therefore, may not be an absolute marker of rejection [34]. Moreover, it is important to emphasize that our results are limited to the innate immune response, and do not evaluate the involvement of the acquired immune system that includes B- and T lymphocytes and their reaction towards dUA, as well as antibodies against the implants [12,35]. However, macrophages are considered to be the key first elements in the immune rejections against decellularised scaffolds and will also respond to residual DNA fragments remaining after decellularisation [34]. Since the majority of the infiltrated cells in the implants herein indeed were CD68^+^, it also seems reasonable to speculate that the acquired immune response against dUA is limited. However, CD45/CD68 double stain is needed to verify the extend of infiltration of macrophages and future studies using B- and T-cell markers are required to confirm these speculations of involvement of the acquired immune system. Moreover, it seems valuable to further profile the extent of dUA tissue remodelling, since the M2b macrophage subtype secrete pro-inflammatory cytokines, such as TNF-α and IL-1β, whereas subtype M2a and M2c are associated with tissue remodelling only [23].

In summary, we found that decellularised human umbilical artery elicits a minimal pro-inflammatory host response both in vivo and ex vivo further emphasizing its potential as a TEVG for bypass in humans.

## 4. Materials and Methods

### 4.1. Approval

The use of human derivatives was approved by The Regional Committees on Health Research Ethics for Southern Denmark (# S-20160181), whereas all animal experiments were licensed by the Danish Animal Experiments Inspectorate (# 2016-15-0201-00941) according to the directive 2010/63/EU for the care and use of laboratory animals. The study was conducted according to the guidelines of the Declaration of Helsinki.

### 4.2. Decellularised Human Umbilical Artery Scaffolds

Human umbilical arteries (UAs) were dissected from fresh human umbilical cords, obtained from Odense University Hospital, while stored in 0.9% NaCl supplemented with 50 IU/mL heparin (5000 IU/mL, Amgros, Copenhagen, Denmark). All enrolled subjects gave written informed consent before donation of the umbilical cord. The UAs were decellularised by immersion and perfusion with various solutions at room temperature for 76 h in total. The procedure consisted of a 16-h rinse in sterile water supplemented with 0.05% sodium azide (NaN_3_; Sigma-Aldrich, St. Louis, MO, USA, cat. No.: S2002), refreshing the solution after 1 h. The UA was then treated for 24 h with a sterile filtered detergent solution (4% sodium deoxycholate (Sigma-Aldrich, cat. No.: 30970) and 1% Triton X-100 (Sigma-Aldrich, cat. No.: T8787)) supplemented with 0.05% NaN_3_, and followed by a 12-h rinse in sterile water supplemented with 0.05% NaN_3_. Finally, the UA was treated with a DNase solution (4 mg/L DNase, Sigma-Aldrich, cat. No.: DN25) and 0.2 mM MgCl2·6H2O (Calbiochem, Sigma-Aldrich, cat. No.: 442611) diluted in sterile water) for 12 h, before rinsing in phosphate-buffered saline (PBS) supplemented with 0.05% NaN_3_ for 12 h. The dUA was then stored in PBS supplemented with 0.05% NaN_3_ at 4 °C, until further use. All perfusions were performed using a peristaltic pump (Ismatec REGLO analog, Ismatec, Wertheim, Germany, cat. No.: MS-4/6) at 90 rpm, with peristaltic tubing (Tygon LMT-55, Ismatec) exhibiting an 0.51 mm internal diameter and a wall thickness of 0.91 mm, resulting flow rate in 1.71 mL/min or 102.6 mL/h. All immersions were performed on a shaker table at 90 rpm.

### 4.3. Animals

Twenty-seven 10-week-old female C57BL/6J (Taconic, Ejby, Denmark) mice were included in the study. They were fed ad libitum with Altromin 1324 pellets (11% fat, 24% protein, 65% carbohydrates, Brogaarden, Lynge, Denmark, cat. No.: 30404) and had free access to water.

### 4.4. Subcutaneous Implantation of dUA into Mouse

Mice were randomly assigned to each of the three experimental groups (1) dUA patch (*n* = 12), (2) Sham (*n* = 8), and (3) Mock (*n* = 7). Subcutaneous implantation was performed for dUA patches (5 mm × 5 mm) on the dorsal right side of the mouse, one per mouse. Sham represented the exact procedure without insertion of a dUA patch, the mock control included no surgery at all. In brief, the mice were weighed and for anaesthesia, a solution of 10 mg/mL ketamine (MSD Animal Health, Kenilworth, NJ, USA) and 1 mg/mL xylazine (Bayer, Leverkusen, Germany) was prepared. The mice were anaesthetised with 0.1 mL ketamine and xylazine mixture per 10 g body weight and placed on a cabinet heated to 25 °C. The effect of anaesthesia was checked by pinching the mouse to test for reflexes. The neck and shoulder region of the mouse was shaved free of hair and wiped off with 70% ethanol. A drop of Viscotears (2 mg/g, Alcon, Novartis, Basel, Switzerland) was applied to each of the mouse’s eyes to prevent drying during surgery. The dorsal skin was opened for about 5 mm along the thoracic spine. A small subcutaneous pocket on the right side was made by blunt separation using scissors. For the sham group, the wound was then immediately closed using 5-0 Ethilon sutures (Ethicon Inc., Somerville, MA, USA, cat. No.: EH7823). For the dUA group, a 5 × 5 mm piece of dUA patch was inserted into the pocket with the abluminal side of the vessel facing towards the skin of the mouse, followed by closure of the wound with 5.0 suture. For analgesia, 0.03 mg/mL buprenorphine (Indivior, Richmond, VA, USA) in saline at a dose of 0.1 mL buprenorphine solution per 10 g body weight was administered, and the animal was put in a cage for awakening in a cabinet heated to 25 °C.

### 4.5. Blood Collection

Venous blood samples were taken 7 days before and 1, 4, 7, 14, 21, and 28 days after the surgery. The blood samples were analysed for white blood cell- and monocyte counts by a scil Vet abc haematology analyser (scil animal care company GmbH, Viernheim, Germany).

### 4.6. Chemiluminescence Analysis

Plasma was isolated from each blood sample, pooled randomly from two to four mice from the same group to achieve enough sample material. They were then analysed for inflammatory cytokines (KC/GRO, IFN-γ, IL-2, TNF-α, IL-1β, IL-5, and IL-10) using the V-PLEX Proinflammatory Panel 1 Mouse Kit (Meso Scale Discovery, Rockville, MD, USA, cat. No.: K15048D-1) according to the manufacturer’s recommendations. The Kit was read on a SECTOR Imager 6000 plate reader (Meso Scale Discovery) according to the manufacturer’s instructions. The lower limit of detection (LLOD) was a calculated concentration based on a signal 2.5 standard deviations above the blank (zero) calibrator.

### 4.7. Explantation

At day 28 post-surgery, the animals were sacrificed and the implanted dUA and underlying tissue was explanted together with skin, by performing a small incision above the implant, and cutting a square around the implant with scissors. For sham animals, skin and subcutaneous tissue samples were taken at the pocket location. For the mock animals, skin and subcutaneous tissue samples were taken from a location similar to that of the other two experimental animal groups. Dissected tissue was embedded in a Tissue-Tek O.C.T. compound (Sakura, Tokyo, Japan, cat. No.: 4583), snap-frozen in dry ice-cooled isopentane and stored at -80 °C until cryosectioning. For sectioning, cryoembedded tissue samples were at sectioned in 10 µm thick sections and stored at −80 °C until staining.

### 4.8. Histology, Immunohistochemistry, and Image Analysis

Sections were fixed in 10% neutral buffered formalin (NBF; Sigma-Aldrich) and stained with haematoxylin and eosin (H&E; Sigma-Aldrich), Miller’s Elastic Van Gieson stain (Atom Scientific Ltd., Cheshire, UK), or immunofluorescence visualizing the DNA by DAPI (Vectashield, Vector Laboratories, Burlingame, CA, USA, cat. No.: H-1200). For immunofluorescence, sections were stained with one of the following primary antibodies: rat anti-CD45 (1:50, BD Biosciences, Franklin Lakes, NJ, USA, cat. No.: 550539), rabbit anti-CD68 (1:500, Abcam, Cambridge, UK, cat. No.: ab125212), rabbit anti-TNFα (1:200, Thermo Fisher Scientific, Waltham, MA, USA, cat. No.: P350), rabbit anti-CD206 (1:2000, Abcam, cat. No.: ab64693), and rat anti-CD31 (1:50, BD Biosciences, cat. No.: 553370), visualised with Alexa Fluor-488 rat or rabbit secondary antibody (1:200, Molecular Probes, Eugene, OR, USA, cat. No.: A21208 and cat. No.: A21206) and mounted with DAPI. Stained tissue sections were examined and imaged using a Leica DMI 4000 B microscope with a Leica CTR4000 illuminator and Leica DFC300FX/DFC 340 FX cameras. The H&E images were analysed by ImageJ (version 1.52a, National Institutes of Health, Bethesda, MD, USA) for the recellularised area and mean greyscale value. In details, the H&E images were quantified first for the area infiltrated by host cells. Then the images were converted to greyscale images and the total explant were marked and measured for the average greyscale value.

### 4.9. Decellularised UA Lyophilization and Pulverization

Decellularised umbilical artery scaffolds were thoroughly washed with PBS for 4 h. They were then lyophilised (Heto Maxi-Dry Plus, Heto-Holten, Allerød, Denmark) for three rounds of 10 h with a 1 h refreeze at −80 °C in between rounds. Lyophilized dUA were then pulverised for three rounds of 5 min at 30 Hz in a TissueLyser (Retsch MM301, Qiagen, Hilden, Germany) using stainless-steel beads (0.5 mm in diameter) and snap freezing for 10 min between rounds.

### 4.10. Macrophage Polarization Assay

Human Peripheral Blood Mononuclear Cells (PBMCs) were isolated by density gradient centrifugation from whole blood (*n* = 3) upon written consent. The monocyte fraction was then isolated from PBMCs using EasySep Human Monocyte Enrichment Kit without CD16 depletion (Stemcell Technologies, Vancouver, BC, Canada, cat. No.: 19058). Monocyte enrichment was assessed by relative qRT-PCR (see Section 4.11) and flow cytometry (LSRII flow cytometer; BD Biosciences). Briefly for flow cytometry, PBMCs and negatively selected monocytes were stained with FITC Mouse Anti-Human CD14 (BD Biosciences, cat. No.: 561712) and Alexa Fluor^®^ 647 Mouse Anti-Human CD16 (BD Biosciences, cat. No.: 561724). Gates were defined using the same samples stained with FITC Mouse IgG2a κ Isotype control (BD Biosciences, cat. No.: 555573) and Alexa Fluor^®^ 647 Mouse IgG1 κ Isotype control (BD Biosciences, cat. No.: 557714). Data analysis was performed using the FACSDiva software (Version 8.0.1, BD Biosciences).

Enriched monocyte fractions were seeded at 280,000 cells/cm^2^ in serum-free macrophage differentiation medium (Stemcell Technologies, cat. No.: 10961) supplemented with human recombinant M-CSF (Stemcell Technologies, cat. No.: 78150) at a final concentration of 50 ng/mL (37 °C; 5% CO_2_). At day 4, cultured cells were activated for 2 days with macrophage differentiation medium/M-CSF further supplemented with lyophilised dUA or nUA (10 mg/mL), IL-4 (10 ng/mL, Sigma-Aldrich, cat. No.: SRP4137) for M2 polarization, or a M1 positive control cocktail containing LPS (10 ng/mL, Sigma-Aldrich, cat. No.: L3012) and human recombinant IFNγ (50 ng/mL, Sigma-Aldrich, cat. No.: IF002). At day 6, RNA was harvested in the culture plates using TRI Reagent solution (Thermo Fisher Scientific, cat. No.: AM9738).

### 4.11. Relative Quantitative Real-Time PCR (qRT-PCR)

Total RNA was isolated from lysed cells and then reverse transcribed into cDNA using a High-Capacity cDNA Reverse Transcription Kit (Applied Biosystems, Waltham, MA, USA, cat. No.: 4368814). Relative quantitative RT-PCR was performed on the QuantStudio 7 Flex platform (Applied Biosystems) using the Power SYBR green PCR master mix (Applied Biosystems, cat. No.: 4367659). Specific forward and reverse qRT-PCR primers were as follows: 5’-GAAGACTTATCGACCATGGAGC-3’ and 5’-AGACGCAGCGGAAATCTTCA-3’ for human CD14; 5’-CTCCCAACTGCTCTGCTACT-3’ and 5’-CGAGCACCCTGTACCATTGA-3’ for human CD16; 5’-CTCCTTGTCATTTTCCAGGTA TGC-3’ and 5’-AAAGTTCCGCACGTCCTTCT-3’ for CCR7; 5’-TCTGAGCCTACAGCAG AGGA-3’ and 5’-AGAGAGGTACTCCTTGAATGCC-3’ for CXCL10; CD80; 5’-TCGCCA GTGAAATGATGGCT-3’ and 5’-TGGAAGGAGCACTTCATCTGTT-3’ for IL-1β; 5’-AGAG GCACTGGCAGAAAACA-3’ and 5’-TCACCAGGCAAGTCTCCTCA-3’ for IL-6; 5’-TAGCC CATGTTGTAGCAAACCC-3’ and 5’-ATCTCTCAGCTCCACGCCATT-3’ for TNFα; 5’-GTC GTACGCTGTGAAGGCAT-3’ and 5’-GGAAAGCCAGGTACTTCAACTT-3’ for RPL13a; 5’-CATTCTCGCTAACAACTGCCC-3’ and 5’-TGATGGACACCAGTTTTAGCCA-3’ for RPL30; 5’-CGACATGGCCAAACGTACCA-3’ and 5’-CAAGTGTACTTGGCGTGCTG-3’ for RPL37a. Data were normalised against several stably expressed endogenous control genes according to the qbase+ platform (version 3.2, Biogazelle, Zwijnaarde, Belgium).

### 4.12. Statistical Analysis

Each analysis consisted of at least three independent experiments designated n. In some experiments, replicate measures, such as those representing dUA from the same donor, were assigned n*. Statistical analysis was performed as indicated throughout the main text and figure legends. Significance level was set at α = 0.05, and the GraphPad Prism (9.0a Mac version; GraphPad Software, San Diego, CA, USA) software was used for all statistical calculations. *p* values are indicated with asterisk (* *p* < 0.05, ** *p* < 0.01, *** *p* < 0.001, **** *p* < 0.0001).

## Data Availability

The data that support this study are available from the corresponding author upon reasonable request.

## Supplementary Materials

The following are available online at https://www.mdpi.com/article/10.3390/ijms22157981/s1.
