# Supplementary material for: Decellularised Human Umbilical Artery as a Vascular Graft Elicits Minimal Pro-Inflammatory Host Response Ex Vivo and In Vivo
Source: Int J Mol Sci. 2021 Jul 26;22(15):7981. doi: 10.3390/ijms22157981 (PMC8347020; doi:10.3390/ijms22157981)
Supplement: Supplementary file 1 [file ijms-22-07981-s001.zip › ijms-1298505-supplementary.pdf]

Title:

Decellularised Human Umbilical Artery as a Vascular Graft Elicits Minimal Pro-inflammatory Host Response *Ex Vivo* and *In Vivo*

Alexander Høgsted Ahlmann [1,2,*], Shu Fang [1,2,*], Sussi Bagge Mortensen [2,3], Line Weis Andersen [2,3], Pernille Gejl Pedersen [1,2], Johanne Juel Callesen [1,2], Sara Thornby Bak [1,2], Kate Lykke Lambertsen [4,5,6] and Ditte Caroline Andersen [1,2,#]

[1] DCA-Lab, Department of Clinical Biochemistry and Pharmacology, Odense University Hospital, J. B. Winsløwsvej 25, 1st floor, 5000 Odense C, Denmark; aahlmann@health.sdu.dk (A.H.A.); sfang@health.sdu.dk (S.F.); pgpedersen@health.sdu.dk (P.G.P.); jojujensen@health.sdu.dk (J.J.C.); sabak@health.sdu.dk (S.T.B.)

[2] Institute of Clinical Research, University of Southern Denmark, J. B. Winsløwsvej 19, 5000 Odense C, Denmark; Sussi.Bagge.Mortensen@rsyd.dk (S.B.M.); Line.Weis.Andersen@rsyd.dk (L.W.A.)

[3] Department of Clinical Immunology, Odense University Hospital, J. B. Winsløwsvej 4, 5000 Odense C, Denmark

[4] Department of Neurobiology, Institute of Molecular Medicine, University of Southern Denmark, J.B. Winsløwsvej 21, 5000 Odense C, Denmark; klambertsen@health.sdu.dk (K.L.L.)

[5] Department of Neurology, Odense University Hospital, J.B. Winsløwsvej 4, 5000 Odense C, Denmark

[6] BRIDGE—Brain Research—Inter-Disciplinary Guided Excellence, Department of Clinical Research, University of Southern Denmark, J.B. Winsløwsvej 4, 5000 Odense C, Denmark

# Correspondence: dandersen@health.sdu.dk; Tel.: +45-65503975

* These authors contributed equally to this work

Supplementary figures

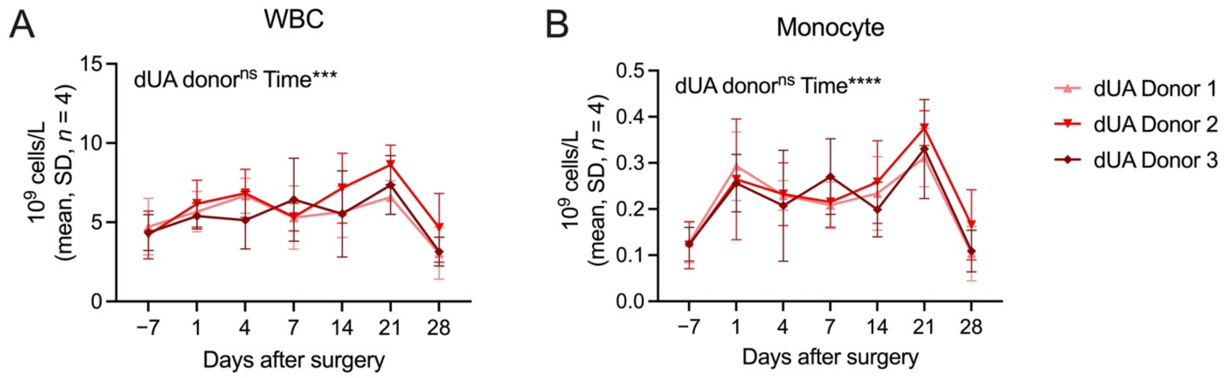

Figure S1. Overall systemic immune response upon dUA implantation in mice.
(**A**) White blood cell (WBC) count and (**B**) monocyte count measured for each test group (dUA donor 1, 2 and 3) 7 days before surgery and 1, 4, 7, 14, 21, and 28 days after surgery. Mixed-effects analysis (post-test: Tukey test) was performed for statistics (n=4). For both WBC and monocyte count, there are no significant differences among different dUA donor groups and data was thus pooled together as one group and used in Figure 1D in the main text.

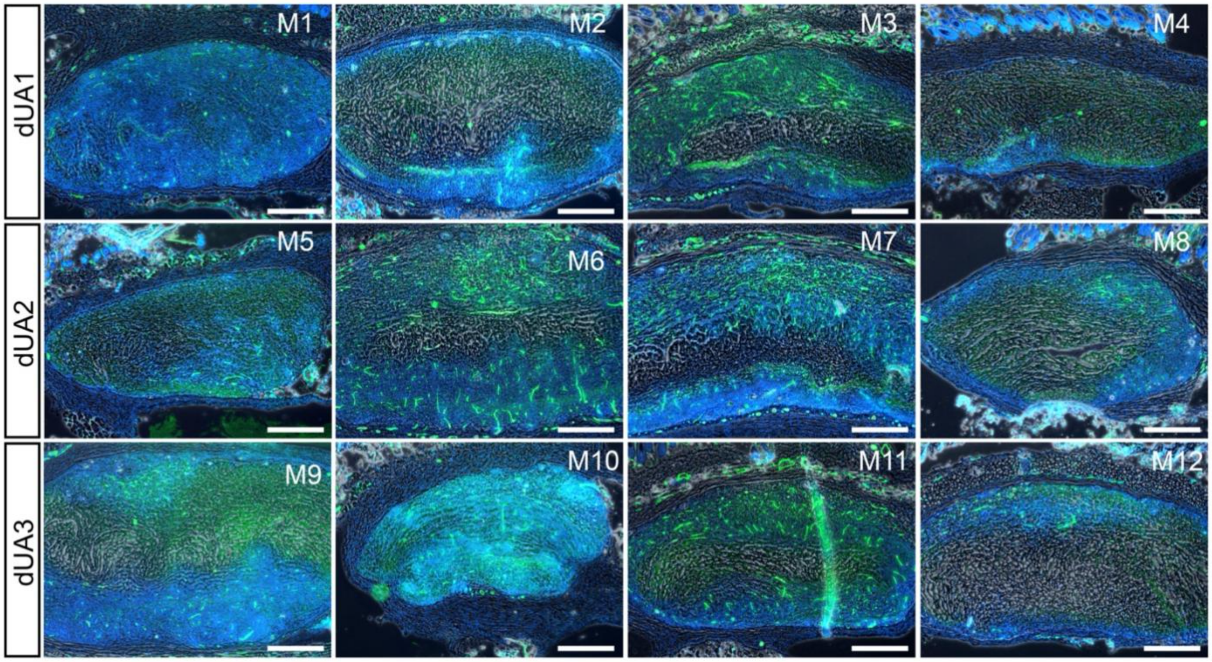

Figure S2. Identification of endothelial remodelling inside the dUA upon *in vivo* implantation.
Representative images from fluorescent staining of the endothelial marker CD31 in all dUA explants (mouse individuals: M1-M12). Scale bars = 500 μm.

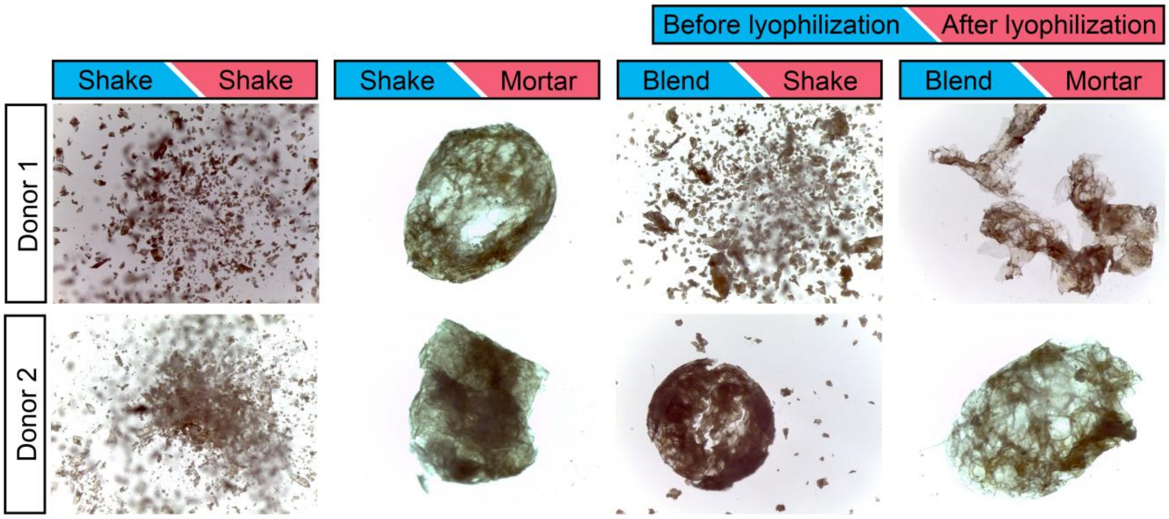

Figure S3. Optimisation of dUA powdering methods for *ex vivo* stimulation of human macrophages.

(A) Microscopic analysis of pulverisation methods before and after lyophilisation. dUA was lyophilised and pulverised through a combination of three different methods: shaking the samples with steel beads, crushing the samples with mortar and pestle, and blending the samples. The most effective pulverisation was seen in the combination of shaking the samples with steel beads both before and after the lyophilisation.

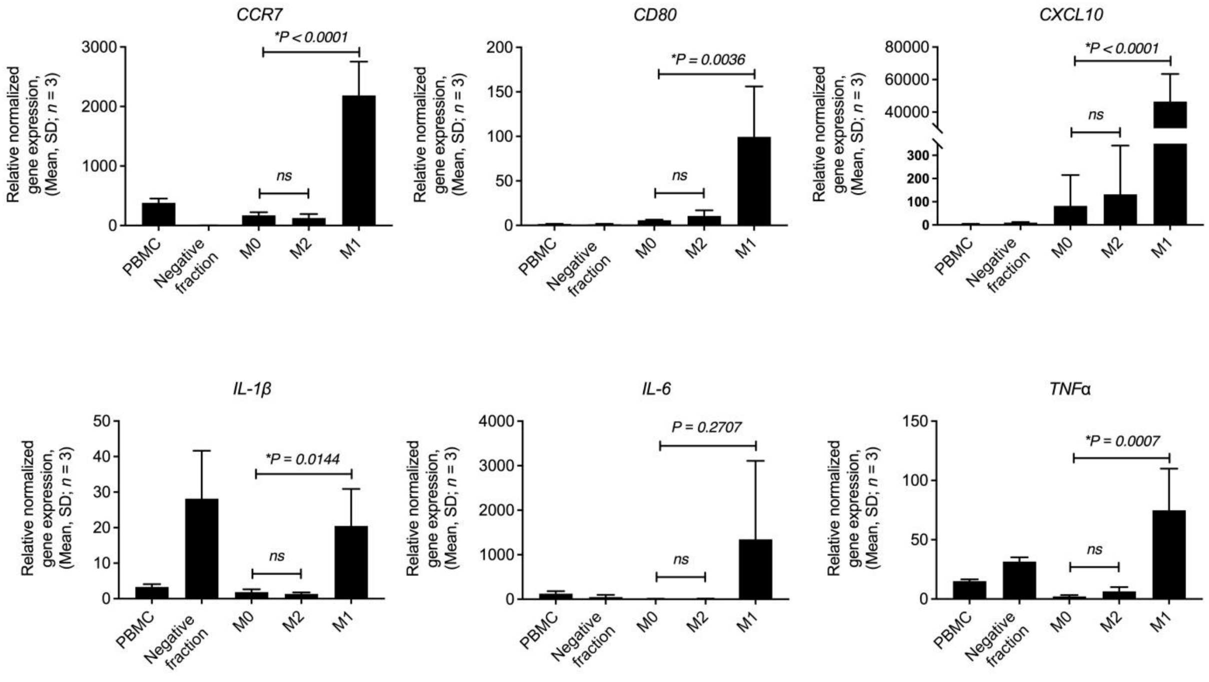

Figure S4. M1 macrophage specific gene expression.

PBMCs and negative selected monocytes as well as differentiated macrophage descendants (M0, M1 and M2) were analysed by qRT-PCR for expression of M1 markers suggested in the literature. Three independent experiments based on three different donors were run. Ordinary one-way ANOVA (post-test: two-stage step-up method of Benjamini, Krieger and Yekutieli) was used for statistical analysis (n = 3), and qRT-PCR raw data were normalised against multiple stably expressed endogenous controls according to the qbase+ platform (data not shown).
